# Healthcare responding to violence and abuse in Brazil: a quasi-experimental difference-in-differences analysis

**DOI:** 10.1016/j.lana.2025.101114

**Published:** 2025-05-08

**Authors:** Estela Capelas Barbosa, Stephanie Pereira, Loraine J. Bacchus, Manuela Colombini, Gene Feder, Lilia Blima Schraiber, Ana Flávia Pires Lucas d'Oliveira

**Affiliations:** a Department of Population Health Sciences, Bristol Medical School, University of Bristol, Canynge Hall, 39 Whatley Road, Bristol, BS8 2PS, UK; b Department of Preventive Medicine, School of Medicine, University of São Paulo, Av. Dr. Arnaldo, 455 Cerqueira César, São Paulo, 01246-903, Brazil; c Department of Global Health and Development, Faculty of Public Health and Policy, London School of Hygiene & Tropical Medicine, 15-17 Tavistock Place, London, WC1H 9SH, UK

**Keywords:** Domestic violence, Violence against women, Primary healthcare, Intervention, Low and middle income countries

## Abstract

**Background:**

Domestic violence against women (DVAW) is a public health issue and a breach of human rights, yet evidence on effective interventions remains limited, particularly in low-income and middle-income countries. This study aimed to evaluate changes in identification and referral to specialist support associated with system-level strategies implemented within Brazilian primary healthcare (PHC) to strengthen the response to DVAW. The strategies comprised an intervention called Healthcare Responding to Violence and Abuse (HERA).

**Methods:**

Using a quasi-experimental design, HERA was implemented in eight PHC clinics, while 33 served as controls. Data on DVAW identification and referral were obtained from the national Epidemiological Surveillance System. Difference-in-differences analysis, using negative binomial regression, assessed HERA's impact, controlling for patient inflow, clinical supervision, COVID-19 lockdown, region, and clinic. Results are reported as marginal effects with 95% confidence intervals (CI).

**Findings:**

There was an increase in the probability of DVAW identification (0.47; 95% CI 0.18–0.77) and referral to support services (0.38; 95% CI 0.03–0.73), when adjusting for panel effects and time. The results were even larger when further controlling for additional variables (0.82 for identification [95% CI 0.44–1.21] and 0.87 for referrals [95% CI 0.47–1.29]).

**Interpretation:**

HERA strategies increased DVAW identification and referral in PHC settings. Clinics implementing HERA were already more likely to identify and refer cases before the implementation, suggesting that HERA's strategies may be more effective in clinics that find DVAW interventions more acceptable, at least in Brazil.

**Funding:**

NIHR Global Health Research Group Award.

**Research in context**

**Evidence before this study**

We searched PubMed and LILACS in March 2025 using the terms (“domestic violence” OR “intimate partner violence” OR “violence against women” OR “gender-based violence”) AND “primary healthcare” AND “intervention” AND “evaluation.” Our search aimed to identify studies evaluating interventions addressing domestic violence against women (DVAW) within primary healthcare (PHC) settings. Our search identified 17 relevant studies. The literature indicates considerable challenges in addressing domestic violence. While a growing body of evidence suggests that targeted strategies can enhance PHC responsiveness, most studies have been conducted in high-income countries, limiting their generalisability to low-income and middle-income countries (LMIC). Thus, there is an urgent need for robust evidence on effective implementation strategies tailored to the unique circumstances of LMIC.

**Added value of this study**

To our knowledge, this is the first Brazilian study using a quasi-experimental design to evaluate a system-level set of implementation strategies to DVAW. It provides compelling evidence for the effectiveness of culturally tailored strategies aimed at improving the response of PHC settings to DVAW. The findings reveal significant improvements in both identification and referral rates, even after accounting for variables such as patient inflow. This evidence not only underscores the intervention's potential but offers a valuable framework for policymakers and healthcare providers seeking to implement effective strategies in similar contexts.

**Implications of all the available evidence**

Findings can inform public policy and guide healthcare managers in implementing best practices for addressing DVAW. By demonstrating the effectiveness of implementation strategies in PHC, this evidence underscores the need to integrate such strategies into broader public health initiatives. It serves as a critical step toward scaling the implementation nationally, ensuring that more women gain access to essential support services and enhancing the capacity of healthcare providers to assist survivors, ultimately improving health outcomes.

## Introduction

Domestic violence against women (DVAW) is a global public health issue and a breach of human rights.^1^ While some progress has been made in the regulatory space to respond to DVAW within the health services,^2^ evidence of effective interventions is still sparse, particularly in low-income and middle-income countries (LMICs).^3^ Globally the prevalence of DVAW varies between 15% and 71%,^4^ with LMICs being at the higher end of this spectrum.^5^

Previous studies on this topic have highlighted the health impact of DVAW, including the associations between violence exposure and mental and physical health problems, increased likelihood of sexually transmitted diseases (including HIV), unplanned pregnancy and its termination and other gynaecological issues.^6^^,^^7^ While one-off interventions are unlikely to produce a sustainable positive responses to DVAW, previously published reviews have highlighted some DVAW interventions in healthcare settings that were effective^3^ and cost-effective.^8^ Furthermore, they reinforce the importance of health-care facilities as key locations for implementing interventions.

In Brazil, around one third of women have reported current or previous experiences of DVAW, especially perpetrated by intimate partners.^5^ A prevalence study conducted in public health services of São Paulo city and its metropolitan region found an even higher prevalence among women service-users, with 76% reporting any type of violence and 55% reporting physical and/or sexual violence.^9^ Brazil has a comprehensive policy framework on DVAW and São Paulo city, where this study took place, has had specific health sector policies since 2002. However, their implementation has been piecemeal and low priority.^10^

The HERA (Healthcare Responding to Violence and Abuse) Programme was an international collaboration, involving research partners in the UK, Brazil, Sri Lanka, Nepal and Palestine. Amongst its objectives, the programme aimed to strengthen the healthcare system response to DVAW, in order to ultimately ensure better outcomes for women and children. This study evaluates the effectiveness of the Brazilian branch of HERA study–a system-level set of implementation strategies which aimed to strengthen the primary care services of the Brazilian Universal Health System (Sistema Único de Saúde—SUS)–in increasing the identification of DVAW and referral to support services. The study explores before and after effects using difference-in-difference techniques in a quasi-experimental design, using observational data.

## Methods

### Study design and settings

This study used a before-and-after quasi-experimental design, using observational data, and was based within primary healthcare (PHC) services in São Paulo, the largest and most populous city in Brazil. Two regions (West and South) were selected to participate in the research. The research team conducted a formative evaluation to assess the readiness of clinics to determine their inclusion in the implementation arm. Thus, clinics that were more willing and better prepared were recruited first, although there was a variation in readiness between them. Four clinics in each region were selected as implementation clinics (8 in total). Instead of using a matched-control design (matched at clinic level), we chose to allocate all other (non-recruited) clinics in the regions of West and South to the control arm (11 in West region and 22 in South region). This resulted in a final sample of 41 clinics (8 implementation and 33 control). This approach allowed for comparison with a larger number of non-recruited clinics, increasing the power of our statistical analyses. Due to the quasi-experimental nature, no power calculation took place before this study. Since the study areas were restricted to South and West of the metropolitan area of Sao Paulo, there is no reason to believe that the populations in the implementation and control arm differ epidemiologically.

There are several reasons why it was not possible to conduct a cluster randomised controlled trial (cRCT). First, the programme aimed to systematically evaluate the integration of HERA into primary care settings in São Paulo city (Brazil) as it was already implemented in the real world. For the control arm, this meant that no specific pathway of referral was in place when DVAW was identified, resulting in a lack of support for victims of abuse in the control arm. The absence of an alternative intervention made randomisation unethical. Second, due to difficulties in implementation and concerns around spillover effects. We relied on observational data for this quasi-experimental difference-in-difference and there was no additional information available (other than patient inflow) which we could have used to balance the clinic's characteristics. Third, the very high turnover of healthcare professionals would result in substantial contamination between implementation and control. Finally, a full cRCT would have been prohibitively expensive and even more difficult to implement, especially during the COVID-19 pandemic.^11^^,^^12^ Given that the implementation strategy is delivered at the level of the clinic (not the individual clinician), the unit of randomisation would have to have been the clinic. So, a fully powered randomised controlled trial would have required dozens of clinics in each arm. This was not feasible within the resources of our research programme nor necessary for evaluating the feasibility and acceptability of the implementation strategy. Finally, given the absence of other initiatives to improve the clinic response to DVAW in Sao Paulo primary care, it is unlikely that the implementation outcomes are due to a secular trend throughout the primary care system.

### HERA implementation strategies

HERA comprises a set of implementation strategies to strengthen the current municipality policy, which requires that each health service should have a Violence Prevention Nucleus (Núcleo de Prevenção à Violência–NPV) composed of at least four healthcare providers and the facility manager. The NPV is responsible for in-service training, epidemiological surveillance, support for violence cases within the health sector, and coordination with a specialised multi agency network DVAW. The implementation research included a formative evaluation phase aimed at identifying obstacles and facilitators to addressing DVAW in PHC services.^13^^,^^14^ The development of the implementation strategies was established through stakeholder meetings with various levels of municipal managers, NPV members and other key representatives. This development process is described in more detail elsewhere.^15^

The HERA implementation strategies were pilot tested, demonstrating its feasibility and acceptability among healthcare providers and women.^16^ HERA comprised a set of implementation strategies: (i) establishment of a referral pathway for the identified cases within the primary healthcare service; (ii) 12 h training to NPV members to strengthen their role; (iii) 4 h general training to all the staff in the PHC clinics to enhance identification, first line support, documentation and referral according to the established pathway; (iv) development of educational materials; and (v) monthly supervision sessions to NPV members to discuss cases and support their work.

Providers were trained to conduct opportunistic inquiry for domestic violence against women, rather than universal screening. This approach was chosen as the evidence on the benefits of universal screening for domestic violence is inconclusive.^17^ No formal screening tools were used, as our model emphasized clinical judgment and context-specific inquiry. Healthcare providers were trained to: (i) identify signs and symptoms related to domestic violence against women; (ii) ask about domestic violence in a sensitive, non-judgmental, and confidential manner when these signs were present; and (iii) respond to disclosures with empathetic listening, validation of the woman's needs, and shared decision-making centered on the woman's perspective. After disclosure, providers were trained to offer referrals to internal and external services, ensuring that decisions were made collaboratively and prioritized the woman's safety and preferences.

Based on lessons learned from the pilot study and aiming for sustainable implementation on a larger scale, two adaptations were made. First, the internal referral pathway was adapted to account for differences in organisational cultures within each PHC clinic. Second, the research team trained the NPV members in a ‘train the trainer’ approach enabling them to conduct general training within their own services.

### Recruitment and study registration

Primary care clinics were selected to implement the HERA strategies in the West and South regions of the metropolitan area of São Paulo. The two participant regions were chosen by convenience: the West due to historical projects and partnerships with the University, and the South because managers requested to participate after learning about the study. When negotiating with local managers from both regions to identify clinics for implementing the strategies, we selected clinics based on average size (territory and staff), and prior identification of DVAW cases. In total, four clinics in the West region and four in the South region (eight in total) implemented the HERA strategies. The policy frameworks, health organisational context and the features of the participating clinics are described elsewhere.^18^

The train-the-trainers (T4T) workshop for the West region took place in November 2019, with the roll-out implementation taking place between December 2019 and March 2020. For the South region, T4T workshop was conducted remotely in August and September 2020, due to COVID-19 restrictions. Face-to-face training sessions in the clinics in the South region were held between November 2020 and February 2021.

Ethical approvals were received from the Research Ethics Committee from the University of São Paulo (3.084.387), São Paulo Municipal Health Department (3.150.024), University of Bristol (80,222) and London School of Hygiene & Tropical Medicine (17,114). This study used secondary data obtained from the Epidemiological Surveillance System. The data was fully de-identified and ethical approval did not require individual consent from the women.

### Data sources, measures, and outcomes

Identification and referral data from both implementation and control clinics was obtained retrospectively by extracting data from Brazilian's robust Epidemiological Surveillance System (SINAN), which made reporting cases of violence against women mandatory in 2004. This requirement aims to generate data on identification and referral, serving as indicators for monitoring policy implementation. The research team was not involved in the surveillance or auditing process of SINAN data. This was entirely managed by the PHC clinics as part of the HERA implementation strategy.

When a healthcare provider identifies a case of DVAW, they complete a paper-based notification form. This form is then sent to the Municipal Epidemiological Surveillance Center, where the information is entered into the national SINAN database. The SINAN form includes a section where providers can indicate, using closed questions (yes/no), whether referrals were offered to various services (e.g. health sector, social welfare, justice system, police stations, domestic violence reference centers, among others). While a single woman could be referred to multiple services, referrals were measured by case rather than by the total number of referrals made. This means that each case of domestic violence was counted once, regardless of how many services were offered.

We selected the following variables to extract data, as they were considered to influence practitioners' ability to identify and refer women experiencing violence: patient inflow, clinical supervision, and COVID-19 lockdowns. Patient inflow may have hindered clinicians' capacity to identify and refer, particularly in very busy clinics; this data was provided by the Municipal Health Secretariat. Clinical supervision could have facilitated identification and referral by offering additional support to clinical staff working with patients in violent relationships. COVID-19 lockdown measures may have restricted patients’ ability to seek help in-person at the clinics.^19^ Additionally, we included time, region and individual clinics as controls. Time is necessary in difference-in-difference analyses, whilst region and individual clinics were included to control for possible spillover effects.

We selected the identification of women victims of DV as our primary outcome measure, as the training focused on improving the skills of primary care staff in recognising women exposed to such violence. It is important to note that DV encompassed different types of abuse (e.g.: physical, psychological, sexual or economic) perpetrated by intimate partners or other family members (e.g.: father, brother, son, grandfather, among others).

Referral to specialist support services was our secondary outcome. Both identification and referral are commonly used outcome measures in studies on violence against women and domestic violence.^16^^,^^20^ While they are intermediary non-health outcomes, they are essential for enabling victim-survivors to access the support they need, which ultimately improves their health and wellbeing.

### Statistical analysis

For each outcome (identification and referral), measured in counts, we compared the eight implementation clinics (four per region) to the remaining control clinics in those areas. We considered but decided against individual-level clinic matching for two main reasons. First, we were constrained by data availability and would only have been able to match clinics in terms of their patient inflow, given other patient population characteristics data did not exist. Furthermore, due to the relatively small number of implementation clinics, individual-level clinic matching would have substantially reduced statistical power and potentially introduced selection bias. Instead of matching, we opted for statistical adjustments of (potentially confounding) covariates, such as patient inflow, region, clinic, clinical supervision, and time trends.

We used a difference-in-difference design^21^, ^22^, ^23^ and explored absolute effects of the implementation strategies on outcomes, controlling for panel and time only (baseline models using ordinary least squares (OLS) regressions) and using negative binomial regressions with a logarithmic link function and no offset, to account for overdispersion in the count data for both identification and referral, controlling additionally for patient inflow, clinical supervision, COVID-19 lockdown, region and clinic for a more nuanced understanding of the effects of the implementation strategies. These variables were selected based on programme theory and expert input from the HERA research and implementation group, which included researchers and practitioners with experience delivering similar interventions in LMICs. Mathematical formulation for our models can be found in the Appendix 1. Since the strategies were not implemented simultaneously in the South and West regions, we explored variations by using both calendar time (e.g. Nov/2019) and time in elapsed months (e.g. month 1 post implementation) before and after the implementation. For our baseline OLS models, we assessed normality and homogeneity of variance of residuals assumptions by inspecting residuals and Q–Q plots. These showed no major deviations. While OLS was used for illustrative purposes, our main analysis employed negative binomial regression, which is more appropriate for count outcomes and does not require normality or homoscedasticity of residuals. For the negative binomial models, all models used cluster-robust standard errors at the clinic level to account for repeated observations over time within clinics. We also explored non-linear specifications for patient inflow, including a quadratic term in the negative binomials. While model fit improved slightly (AIC difference >2), the effect size was very small and the non-linear model was more difficult to interpret. Given patient inflow was included as a covariate rather than a primary variable of interest, we kept the linear specification to support simplicity and interpretability.

Our difference-in-differences approach estimates the average treatment effect on the treated (ATET)—that is, the effect of the implementation among clinics that received the intervention. This reflects the design, where only a subset of clinics were exposed to the intervention, and allows us to isolate the implementation effect in real-world settings.

All results are reported as marginal effects, along with 95% confidence intervals and p-values. Marginal effects represent the change in probability of our outcome (identification or referral) associated with the change in time (from before to after), holding all other variables constant. Given our models use a difference-in-difference approach with a negative binomial regression, marginal effects allow for a straightforward interpretation of effect size in absolute terms.

### Role of the funding source

The funder of the study had no role in the study design, data collection, data analysis, data interpretation, or writing.

## Results

### Descriptive characteristics

Table 1 presents the characteristics of the implementation and control practices in each region. While the average patient inflow is similar between regions in the implementation arm, it is lower in the control clinics of the West region and higher in the control clinics of the South Region. More importantly, the difference in number of supervision sessions is large between implementation areas, possibly due to COVID-19 lockdown restrictions that only affected the West region post-implementation. Finally, both regions trained more than two thirds of their staff as part of the programme.

**Table 1:** Relevant characteristics in South and West region clinics by implementation and control arm.

Characteristic	West region		South region	
General	Implementation	Control	Implementation	Control
Number of clinics	4	11	4	22
Average monthly patient inflow	2639	1951	2571	2832
Number of clinical supervision sessions (in 24 months of the study)	9	0	30	0

Important dates	West region	South region
Training of the trainers	November/2019	August and September/2020
Replication at the clinics	December/19 to March/20	November/20 to February/21
Period 12 months before HERA strategies' implementation	November/18 to October/19	September/19 to August/20
Period 12 months after HERA strategies' implementation	November/19 to October/20	September/20 to August/21
Number of months impacted by COVID-19 lockdown	4 in post implementation period	4 in pre implementation period

Training	West region	South region
Total number of staff trained	331	236
% of trained staff	68%	73%

Note: HERA, Healthcare Responding to Violence and abuse.

### Difference-in-difference results

**Fig. 1:**
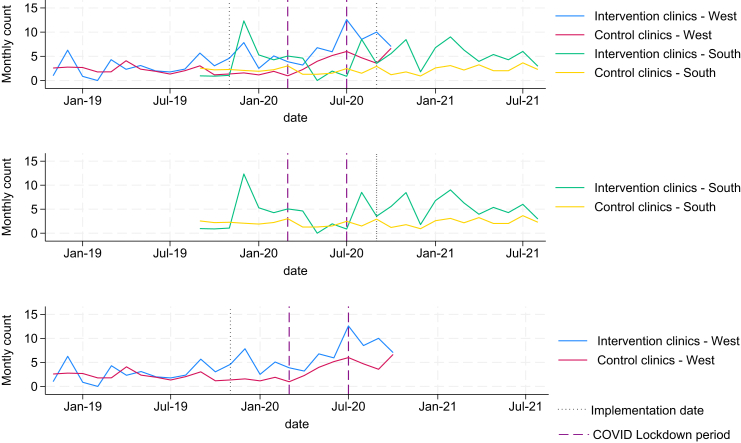
Monthly trends in identification and referral rates by region and intervention status. Note: These plots were used to visually assess the parallel trends assumption underlying the difference-in-differences analysis. While some natural fluctuation is expected, trends appear approximately parallel in the pre-intervention period, particularly in the West region.

Fig. 1 shows the trends in (unadjusted) identification for both West and South regions, plotted over the duration of the study. The first solid line marks the implementation of the first strategy (T4T training) in the West region (November/2019) and the second solid line represents the implementation date for the South Region (August/2020). The COVID-19 lockdown appears in between the dashed lines. The figure shows the similarities in magnitude of identification from both control and implementation clinics in West region pre implementation, and a larger variation in trends between implementation and control clinics in the South region pre-implementation. The figure also shows an increase in the trend of unadjusted identification for both the implementation and control clinics in the South region (although larger in implementation clinics) and a slight reduction in the variation in implementation clinics in the West region, with control clinics trend remaining stable.

Table 2, in turn, presents the actual counts of identification and referrals for the South and West regions, alongside the number of clinics and patient inflow in each group (intervention and control). For all our analyses, the number of counts represents the numerator in the reported proportions, while the patient inflow the denominator in such proportions. When using count models (i.e. negative binomials with logarithmic link function and no offset), we adjust for patient inflow to prevent bias for larger clinics, which might naturally have more identifications and referrals simply because they see more people.

**Table 2:** Before and after counts of identification and referrals (numerators), number of clinics and patient inflow (denominators) for the South and West regions and total.

	Identification								
	Before								
	South			West			Total		
	Counts	N. clinics (n)	Patient inflow	Counts	N. clinics (n)	Patient inflow	Counts	N. clinics (n)	Patient inflow
Implementation	43	4	115,911	38	4	139,393	81	8	255,304
Control	138	22	665,921	62	11	271,071	200	33	936,992

**After**									
Implementation	70	4	130,971	74	4	114,012	144	8	244,983
Control	193	22	829,589	80	11	244,211	273	33	1,073,800

	Referral								
	Before								
	South			West			Total		
	Counts	N. clinics (n)	Patient inflow	Counts	N. clinics (n)	Patient inflow	Counts	N. clinics (n)	Patient inflow
Implementation	41	4	115,911	33	4	139,393	74	8	255,304
Control	127	22	665,921	52	11	271,071	179	33	936,992

**After**									
Implementation	63	4	115,911	66	4	139,393	129	8	255,304
Control	180	22	665,921	74	11	271,071	254	33	936,992

Note: Numerators represent identified or referred cases (counts); denominators correspond to total patient inflow per clinic and period.

The difference-in-difference analysis shows that there was an adjusted increase in the trends of identification and referral to specialist support as a result of the implementation strategies, although for referral, when time was controlled for in calendar month/year, the change was not significant (p = 0.054). The adjusted primary outcome (identification of DVAW) increased between 0.44 and 0.47 (from 81 to 144 identifications), depending on how time was defined. The smaller increase when using time as counted in calendar month/year (e.g. Nov/2019) seems reasonable, because even though the South and West areas are relatively distant from each other, the earlier implementation in the West region may have contributed to a general increase in awareness around victims of violence presenting at primary care across the metropolitan region, so some small contamination may explain the relatively smaller increase. In terms of the secondary outcome, the marginal effects are smaller than those observed for the primary outcome, which is to be expected as it is a necessary condition to be identified to be able to be referred to specialist support. And it is widely known that not all victim-survivors feel able to engage with or want help from support services.^24^ Table 3 summarises the results.

**Table 3:** Difference-in-difference analysis: estimates from regression models looking at change in identification and referrals in West and South regions of Sao Paulo before and after the implementation of the HERA strategies.

Outcome	Marginal effect	95% CI		p-value
Identification (counts per month)				
(time in months–Implementation = T0)	0.47	0.18	0.77	0.0020
(time in calendar month/year–eg. November/19)	0.45	0.14	0.76	0.0050
Referral (counts per month)				
(time in months–Implementation = T0)	0.38	0.03	0.73	0.033
(time in calendar month/year–eg. November/19)	0.35	−0.01	0.71	0.054

Notes: Average Treatment Effect on the Treated (ATET) estimate adjusted for panel effects and time.

CI, Confidence Interval.

HERA, Healthcare Responding to Violence and abuse.

T0, baseline time point.

**Fig. 2:**
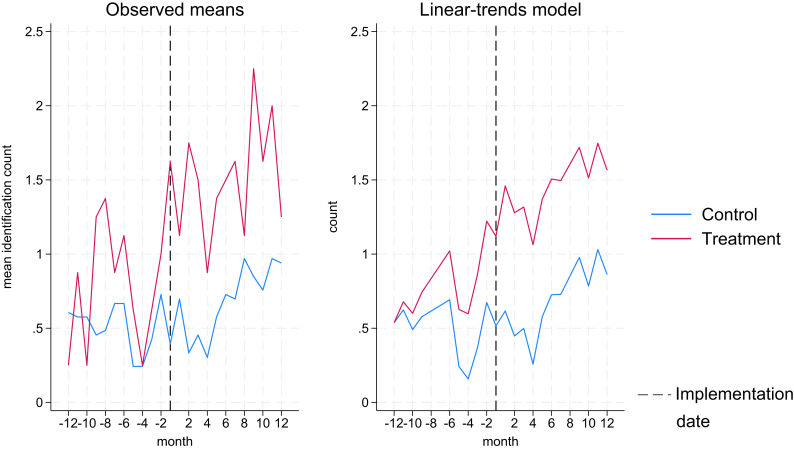
Observed means and linear trends in identification.

When the analysis was performed after negative binomial regressions, additionally controlling for patient inflow, clinical supervision, region, clinic and COVID-19 lockdown, the results show an even greater effect of the implementation strategies. The adjusted probability of identification increased just over 0.82 regardless of how time was accounted for in the models. Furthermore, implementation clinics had a underlying higher probability of identifying patients exposed to violence (0.46); the size of the clinic, proxied by patient inflow was highly significant (p = 0.0001), with larger clinics identifying more patients and, as expected; time (measured in months or calendar months) increasing identification, meaning the underlying trend regardless of the implementation has a positive slope. Fig. 2 shows the trends in identification by observed means and linear-trends model. Table 4 presents the results of two models. Model (1) included time in months (implementation = T0) and model (2) included time in calendar month and year (from Nov/2018 to Aug/2021).

**Table 4:** Difference-in-difference analysis of identification: estimates post negative binomial regressions, additionally controlling for patient inflow, clinical supervision, region, clinic and COVID-19 lockdown.

Variables	(1)			(2)		
	Identification			Identification		
		95% CI			95% CI	
(base before # control)	Marginal effect	Lower bound	Upper bound	Marginal effect	Lower bound	Upper bound
Before # implementation	0.46∗∗∗	0.12	0.81	0.46∗∗∗	0.12	0.81
After # control	−0.20	−0.55	0.16	−0.20	−0.56	0.16
After # implementation	0.83∗∗∗	0.44	1.21	0.82∗∗∗	0.44	1.21
Patient inflow	0.0002∗∗∗	0.0002	0.0003	0.0002∗∗∗	0.0002	0.0003
Supervision	−0.32∗	−0.67	0.037	−0.32∗	−0.67	0.037
Region (base = west)	−0.0082	−0.21	0.19	0.31∗	−0.017	0.64
Clinic	−0.0064	−0.014	0.0015	−0.064	−0.014	0.0015
COVID-19 lockdown	−0.22	−0.53	0.081	−0.22	−0.53	0.081
Time (in months)	0.032∗∗	0.0067	0.057			
Date (from November/18 to August/21)				0.0011∗∗	0.0002	0.0019
Alpha	0.45∗∗∗	0.29	0.69	0.45∗∗∗	0.29	0.69
lnalpha	−0.80∗∗∗	−1.24	−0.37	−0.80∗∗∗	−1.24	−0.37
Constant	−1.36∗∗∗	−1.74	−0.98	−24.34∗∗∗	−42.60	−6.07
Observations	984			984		

Note: CI, Confidence Interval.

∗∗∗p < 0.01, ∗∗p < 0.05, ∗p < 0.1.

**Fig. 3:**
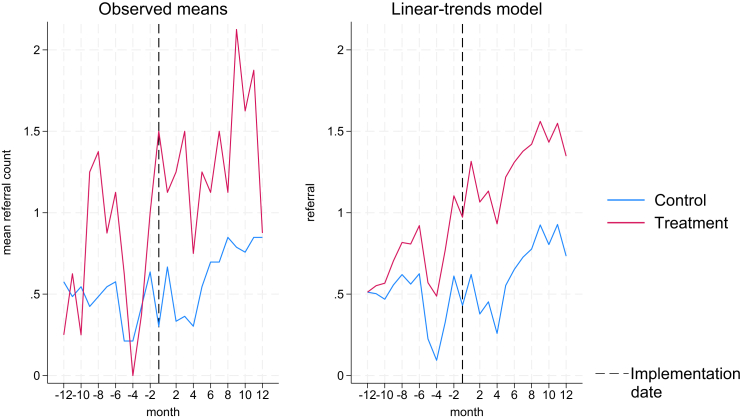
Observed means and linear trends in referral.

In turn, Fig. 3 and Table 5 present the results from the difference-in-difference analysis post negative binomials for our secondary outcome, referrals. Similarly to identification, model (3) uses time in months (implementation = T0) and model (4) includes time in calendar month and year (from Nov/2018 to Aug/2021).

**Table 5:** Difference-in-difference analysis of referral: estimates post negative binomial regressions, additionally controlling for patient inflow, clinical supervision, region, clinic and COVID-19 lockdown.

Variables	(3)			(4)		
	Referral			Referral		
		95% CI			95% CI	
(base before # control)	Marginal effect	Lower bound	Upper bound	Marginal effect	Lower bound	Upper bound
Before # implementation	0.50∗∗∗	0.15	0.85	0.50∗∗∗	0.15	0.85
After # control	−0.12	−0.50	0.26	−0.12	−0.50	0.26
After # implementation	0.88∗∗∗	0.47	1.29	0.88∗∗∗	0.46	1.29
Patient inflow	0.0002∗∗∗	0.0002	0.0003	0.00022∗∗∗	0.0002	0.0003
Supervision	−0.29	−0.68	0.10	−0.29	−0.68	0.10
Region (base = west)	−0.061	−0.27	0.15	0.23	−0.12	0.59
Clinic	−0.0085∗∗	−0.017	−0.0002	−0.0085∗∗	−0.017	−0.0002
COVID-19 lockdown	−0.20	−0.53	0.12	−0.20	−0.53	0.12
Time (in months)	0.029∗∗	0.0026	0.056			
Date (from November/18 to August/21)				0.0009∗∗	0.0001	0.0018
Alpha	0.48∗∗∗	0.31	0.74	0.48∗∗∗	0.31	0.74
lnalpha	−0.74∗∗∗	−1.18	−0.30	−0.74∗∗∗	−1.18	−0.30
Constant	−1.36∗∗∗	−1.75	−0.96	−22.45∗∗	−41.81	−3.09
Observations	984			984		

Note: CI, Confidence Interval.

∗∗∗p < 0.01, ∗∗p < 0.05, ∗p < 0.1.

As can be seen from Table 5, once you control for patient inflow, clinical supervision, region, clinic, and COVID-19 lockdown, as well as panel and time effects, the marginal effects are even larger than those observed for identification. This suggests that the implementation strategies are highly effective for increasing referrals to specialist support services. Similar to the identification models, larger clinics have a larger probability of referring, but unlike models 1 and 2, in models 3 and 4 clinical supervision is not significant (p = 0.14 and p = 0.14 respectively). Additionally, the marginal effect for implementation clinics before the implemented strategies is also larger than that in the identification models, suggesting that implementation clinics not only were already better at identifying victims of abuse, even before the implemented strategies, but they were also more likely to refer those survivors to support services. Table 5 summarises the findings for models 3 and 4. Fig. 3 presents trends of referral counts (observed mean and adjusted linear trend) by implementation group. Appendix 2 presents the results from Tables 4 and 5 as exponentiated coefficients.

## Discussion

In this paper we analysed the effectiveness of a DVAW systems-level set of implementation strategies in primary care in Brazil, using a difference-in-difference design. We found that the HERA implementation strategies were effective in increasing identification and referral of women who experienced violence and presented at primary care clinics. When controlling for panel design and time, the implementation strategies increased the adjusted probability of identification by 0.47 and the adjusted probability of referral by 0.38. When additionally controlling for patient inflow, region, clinical supervision, clinic, COVID-19 lockdown, the adjusted effect of the implementation strategies was even larger (0.82 for identification and 0.87 for referrals), although the implementation clinics were already more likely to identify and refer women patients exposed to violence before the implemented strategies.

Our findings align with those published in a systematic review of violence interventions in sexual and reproductive health settings in LMIC^3^ and reinforce the conclusion that referral of intimate-partner violence cases is a common positively affected implementation outcome.^25^ Findings from related qualitative studies^15^^,^^16^ suggest that the HERA's implementation strategies may have fostered a more woman-centered approach to care within the primary healthcare setting. By prioritising the needs and experiences of women, under a gender and human rights perspective, the strategies may also have enhanced the quality of interactions between healthcare providers and patients.^15^^,^^16^ Clinicians received training in empathetic communication, enabling them to listen actively to women's concerns and provide tailored support that addressed their unique circumstances. Additionally, the increase in referrals reflected a strengthened relationship between the health service and domestic violence specialised services within the multiagency network. This collaboration had potential to, ultimately, ensure that women receive comprehensive and coordinated care, thereby improving their overall experience within the healthcare system, as discussed in a related HERA publication.^16^

Primary healthcare is uniquely positioned to identify and provide support for DVAW due to its accessibility and close community ties. Healthcare providers often develop trusting relationships with patients, which can encourage women to disclose experiences of violence. Also, primary healthcare settings serve as a first and frequent point of contact for many women, enabling early identification of signs of abuse, such as physical injuries or mental health issues related to trauma.^26^ Moreover, the role of community health workers in the implementation outcomes should be further studied, especially regarding referrals, as they play a vital role in Brazilian healthcare services by disseminating information about women's rights, identifying domestic violence cases, and acting as trusted advocates who connect vulnerable populations with essential resources and support.^27^ By integrating comprehensive DVAW responses into routine services, primary healthcare can facilitate timely interventions, offer resources, and connect victims with necessary services, making their journey towards support less critical and time consuming.^28^^,^^29^

This implementation study holds significant implications for clinicians, managers and policymakers. The study demonstrated that ongoing training and resources–including care pathways, committed staff and managers–considerably increased the identification and referral of DV cases by healthcare providers, at least in Brazil. Specific mechanisms such as a gendered and women centred perspective, heightened clinician awareness, streamlined communication with support services, and the establishment of protocols were particularly effective. Clinicians benefited from greater confidence and competence in handling these sensitive issues related to DVAW, which can ultimately lead to improved health outcomes for women.^25^ HERA's implementation strategies also shifted healthcare providers toward a more proactive approach in recognising signs of abuse, enabling timely intervention and support for women. Increased awareness, well-defined referral pathways, and enhanced soft skills created a safer environment for women to disclose their experiences, thereby promoting their rights and ensuring access to vital resources within a multiagency network.^15^^,^^16^

For local policymakers and managers at different levels, the study's implications are profound. The successful outcomes can guide the development of policies, protocols and funding strategies. Our findings underscores the need for comprehensive programs and guidelines that empower healthcare professionals to act decisively. It also highlights the importance of integrating domestic violence protocols into healthcare systems, ensuring every primary care visit can serve as an opportunity for identification and initial support. Ultimately, this study reinforces the critical role of healthcare in addressing domestic violence and advocates for sustained investment in training and resources to improve outcomes for women experiencing domestic violence.

This study is, to the best of our knowledge, the first to use a quasi-experimental design to evaluate a systems-level set of implementation strategies for domestic violence response in primary care settings in Brazil. However, there are a number of limitations to this study. First, this was a pragmatic implementation study, since a cluster randomised controlled trial was not possible due to ethical reasons and funding restrictions. Furthermore, implementation was not randomly allocated, meaning more interested and willing clinics in the west and south regions of São Paulo were more likely to adopt the implementation strategies. This is reflected in our results, which shows those clinics were, a priori, more likely to identify and refer women exposed to violence. We acknowledge that our data does not include information on whether referrals resulted in actual engagement with services. While we attempted to gather this information, coordination across different sectors proved challenging, particularly due to the sensitivity of the data and restrictions on data sharing. The COVID-19 pandemic also impacted the implementation timelines, resulting in a relatively large delay between when the strategies were implemented in the western and southern regions. While this delay allowed for adaptations, it introduced variability in how and when practices were adopted across clinics, which may have faced greater challenges in integrating HERA strategies due to competing demands during the COVID-19 pandemic. In the early phases of the pandemic, clinics were overwhelmed with respiratory cases, which may have reduced the time and capacity available for healthcare providers to conduct opportunistic inquiries about DVAW. Later, as vaccination efforts scaled up, clinics faced additional pressures, further straining their capacity to address other health issues. While we attempted to control for COVID-19 lockdown in São Paulo, these disruptions may have led to underreporting of DVAW cases during certain periods, potentially skewing the results. Another limitation of this study is the lack of reliable information on the ethnicity of domestic violence survivors. While the epidemiological surveillance system collects data on the ethnicity of women, this information is not consistently recorded, as race is self-declared by law in Brazil and often subject to missing data. Due to these inconsistencies and the high proportion of missing data, we chose not to include ethnicity in our analysis. However, we recognize the importance of ethnicity as a potential factor influencing the identification, referral, and outcomes of domestic violence survivors, and its absence represents a limitation of this study. Finally, we deemed a difference-in-difference analysis an appropriate method for our objectives, but acknowledge that it implies a number of assumptions in a quasi-experimental design. One such assumption is that the relatively stable and linear trends observed pre-intervention would have remained in a similar trajectory in the absence of the intervention. Having said that, difference-in-difference methods have been widely regarded as robust in quasi-experimental studies in public health interventions where RCTs are not feasible.^21^

Another limitation of our study was that we did not formally judge the sample size, due to its pragmatic and quasi-experimental nature. However, our main results yielded statistically significant estimates with reasonably narrow confidence intervals, particularly for our primary outcome. This suggests our sample size and design were sufficient to detect meaningful differences, with relatively narrow confidence intervals, supporting the validity of our study. Finally, while we used a difference-in-differences approach rather than a simple before–after comparison, the study remains quasi-experimental using observational data. As such, it is still subject to limitations including regression to the mean, unmeasured time-varying confounding, and potential underlying secular trends that may differentially affect implementation and control clinics over time. We have adjusted for relevant covariates and included clinic and time fixed effects to minimise this, but some residual confounding cannot be completely ruled out.

Future research should explore the generalisability of our findings to other regions in Brazil, considering the particularities of different cultural contexts and healthcare organisations. Given that the implementation strategies were more likely to be adopted by willing clinics in specific regions, it is essential to examine the extent to which selection bias influenced our results. Identifying factors that contributed to the readiness^17^ of clinics to participate and developing strategies to motivate less willing clinics will be valuable for future implementation efforts. Also, investigating the challenges and best practices for scaling up the implementation strategies to a national level will provide insights into its broader applicability and impact. Examining the cost implications of large-scale implementation and identifying effective funding strategies will be important for securing necessary resources. Future studies should also evaluate sustainability of the strategies over a longer timeline and determine how frequently they should be provided or refreshed to maintain high levels of clinician competence and confidence (e.g. training and supervision sessions). Another pertinent area of research involves assessing the long-term outcomes for women identified and referred through the intervention, including whether women engaged with the service they were referred to. Understanding the sustainability of positive effects over time is critical for evaluating the enduring impact of the programme and informing future iterations. Additionally, identifying support systems needed post-referral to ensure the long-term safety and well-being of survivors of domestic violence will be crucial for comprehensive care.

## Contributors

AFO, LJB and GF conceived this study. ECB and SP wrote the initial draft. ECB conducted the data analysis. SP directly accessed and verified the underlying data reported in this study. All authors reviewed, commented on and revised further drafts. All authors had full access to the data in the study and were responsible for the decision to submit for publication.

## Data sharing statement

Clinic-level data used for this study may be shared subject to data governance procedures defined by Universidade de São Paulo. Proposals should be directed to stephaniepereira@usp.br; to gain access, data requestors will need to sign a data access agreement.

## Declaration of interests

Estela Capelas Barbosa and Gene Feder reports salary support from the UK Prevention Research Partnership (Violence, Health and Society; MR-VO49879/1) for the present manuscript. This partnership is funded by the British Heart Foundation, Chief Scientist Office of the Scottish Government Health and Social Care Directorates, Engineering and Physical Sciences Research Council, Economic and Social Research Council, Health and Social Care Research and Development Division (Welsh Government), Medical Research Council, National Institute for Health and Care Research, Natural Environment Research Council, Public Health Agency (Northern Ireland), The Health Foundation, and Wellcome. Gene Feder reports honoraria for clinician teaching events and institutional support from the NIHR for the present manuscript. He also declares travel support from the Global Burden of Disease programme and serves as an unpaid trustee for IRISi. All other authors declare no competing interests.
