# Supplementary material for: Healthcare responding to violence and abuse in Brazil: a quasi-experimental difference-in-differences analysis
Source: Lancet Reg Health Am. 2025 May 8;47:101114. doi: 10.1016/j.lana.2025.101114 (PMC12135433; doi:10.1016/j.lana.2025.101114)
Supplement: Appendix [file mmc1.docx]

# Appendix 1: Statistical Model Specification – Mathematical formulations

## Baseline Model – Ordinary Least Squares (OLS)

Let Y_it_ be the count of identifications (or referrals) in clinic i at time t.

We estimated the baseline Difference-in-Differences (DiD) model using the following specification:

Y_it_ = β₀ + β₁Post_t_ + β₂Treatment_i_ + β₃(Post_t_ × Treatment_i_) + γ_t_ + α_i_ + ε_it_

Where:
- Post_t_ is an indicator for post-implementation period (after)
- Treatment_i_ is an indicator for implementation clinics
- γ_t_ captures time fixed effects
- α_i_ captures clinic fixed effects
- β₃ is the DiD estimate of the implementation effect

## Adjusted Model – Negative Binomial Regression with Log Link and no offset

To address overdispersion in count data, we used a negative binomial regression with the following specification:

log(E[Y_it_]) = β₀ + β₁Post_t_ + β₂Treatment_i_ + β₃(Post_t_ × Treatment_i_) + β₄Inflow_it_ + β₅Supervision_it_ + β₆COVID_t_ + γ_t_ + α_i_

Where:
- E[Y_it_] is the expected number of identifications or referrals
- Covariates include patient inflow, clinical supervision, COVID-19 lockdown,
 and fixed effects for clinic and time
- Marginal effects were derived to interpret changes in probability in absolute terms (percentage points)

# Appendix 2: Negative binomials regression results – exponentiated coefficients – IRR - Models 1 to 4

|  | Model 1 | Model 2 | Model 3 | | Model 4 | |
| --- | --- | --- | --- | --- | --- | --- |
| Variable | IRR | IRR | IRR | IRR | |  |
|  |  |  |  |  | |  |
| before # intervention | 1.59 | 1.59 | 1.65 | 1.65 | |  |
| after # control | 0.82 | 0.82 | 0.89 | 0.89 | |  |
| after # intervention | 2.28 | 2.28 | 2.41 | 2.40 | |  |
|  |  |  |  |  | |  |
| patientflow | 1.0002 | 1.0002 | 1.0002 | 1.0002 | |  |
| supervision | 0.73 | 0.73 | 0.75 | 0.75 | |  |
| Region (base=west) | 0.99 | 1.37 | 0.94 | 1.26 | |  |
| clinic | 0.99 | 0.99 | 0.99 | 0.99 | |  |
| covidlockdown | 0.80 | 0.80 | 0.82 | 0.82 | |  |
| time (in months) | 1.033 |  | 1.030 |  | |  |
| date (from Nov18 to Aug21) |  | 1.0011 |  | 1.0010 | |  |
|  |  |  |  |  | |  |
| /lnalpha | 0.45 | 0.45 | 0.48 | 0.48 | |  |
|  |  |  |  |  | |  |
| constant | 0.26 | 2.70E-11 | 0.26 | 1.78E-10 | |  |
